# Right-sided Cardiac Resynchronization Therapy via Left Bundle Branch Area Pacing in a Patient with Persistent Left Superior Vena Cava

**DOI:** 10.19102/icrm.2025.16054

**Published:** 2025-05-15

**Authors:** Can Menemencioglu, Uğur Canpolat

**Affiliations:** 1 Department of Cardiology, Faculty of Medicine, Hacettepe University, Ankara, Turkey

**Keywords:** Cardiac resynchronization therapy, left bundle branch area pacing, persistent left superior vena cava

## Abstract

Cardiac resynchronization therapy (CRT) via left bundle branch area pacing (LBBAP) has emerged as effective and safe as conventional CRT. Left-sided CRT implantation in patients with persistent left superior vena cava (PLSVC) is challenging and impossible in some patients. Right-sided CRT implantation, either conventional or LBBAP, is also tricky, as the delivery sheaths are feasible for left-sided implantations. Here, we present a patient with PLSVC who underwent successful right-sided CRT implantation via LBBAP.

## Introduction

Heart failure (HF) is one of the most common causes of mortality and morbidity in the world.^1^ In treating heart failure with reduced ejection fraction (HFrEF) due to non-ischemic dilated cardiomyopathy, if there is no improvement in symptoms and ejection fraction despite optimal medical treatment, a cardiac implantable electronic device (CIED) implantation is indicated.^2^ For many years, conventional or biventricular cardiac resynchronization therapy (CRT) via the coronary sinus has been preferred in HFrEF patients with electrical dyssynchrony (QRS duration of >130 ms). As a result of studies conducted in recent years, CRT via left bundle branch area pacing (LBBAP) has emerged as an alternative to conventional biventricular CRT.^3^ Although the left-sided approach is mainly preferred, in some cases (eg, prior left-sided infection, congenital anomalies, venous occlusion), implantation from this region makes the procedure difficult or even impossible. Persistent left superior vena cava (PLSVC) is a challenging anatomical abnormality in left-sided CIED implantation. The data for right-sided CRT via LBBAP in patients with PLSVC^4–8^ are scarce. Herein, we report a right-sided approach for CRT via LBBAP in a patient with PLSVC.

## Case presentation

**Figure 1:**
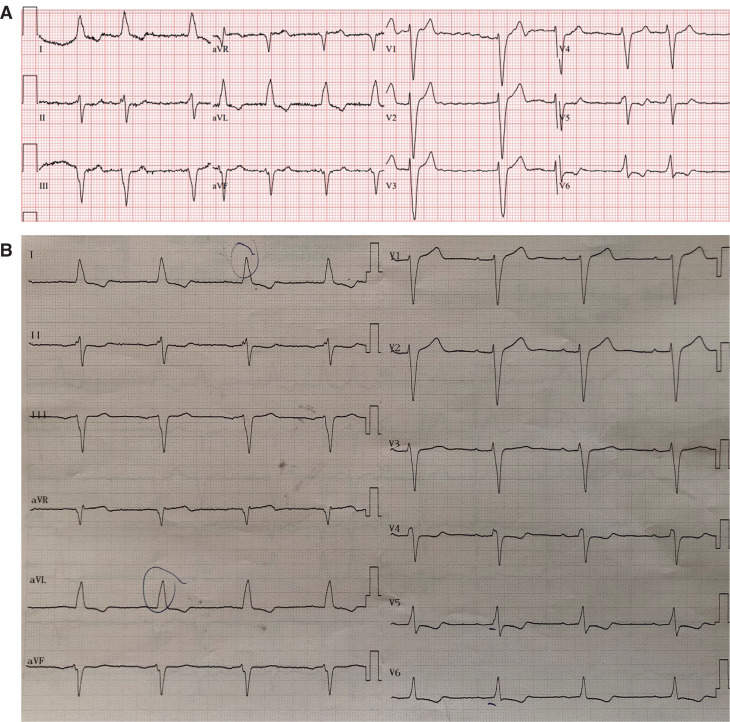
**(A)** The 12-lead electrocardiogram on admission showed atrial fibrillation (78 bpm), a QRS duration of 152 ms, and left bundle branch block (LBBB). **(B)** The sinus rhythm (P–R interval, 240 ms; QRS duration, 160 ms; LBBB) was maintained after facilitated electrical cardioversion using amiodarone infusion.

A 73-year-old woman with a previous history of non-ischemic dilated cardiomyopathy, arterial hypertension, and asthma was admitted to the emergency department with worsened dyspnea for 2 weeks. She had been undergoing optimal HFrEF treatment, including 49/51 mg of sacubitril/valsartan, 6.25 mg of carvedilol, 25 mg of spironolactone, 40 mg of furosemide, and 10 mg of dapagliflozin for 9 months. The physical examination revealed bilateral basal lung crackles, peripheral edema, an irregular pulse, and an apical 2/6 systolic murmur. The 12-lead electrocardiogram (ECG) on admission showed atrial fibrillation (AF) (78 bpm), a QRS duration of 152 ms, and left bundle branch block (LBBB) **(Figure 1A)**. A transthoracic echocardiography (TTE) revealed a dilated left ventricle (LV) (end-diastolic diameter of 58 mm), a reduced LV ejection fraction (LVEF) (25% with the modified Simpson method), global LV hypokinesia, moderate mitral and tricuspid regurgitation, and a left atrial diameter of 41 mm **(Video 1A)**. She was hospitalized for acute decompensated HF treatment. A transesophageal echocardiography excluded the intracardiac thrombus before electrical cardioversion, and the sinus rhythm (P–R interval, 240 ms; QRS duration, 160 ms; LBBB) was maintained after facilitated electrical cardioversion using amiodarone infusion **(Figure 1B)**.

**Video 1:** The TTE after electrical cardioversion demonstrated both septal flash and apical rocking motion besides the LVEF of 31%.

**Figure 2:**
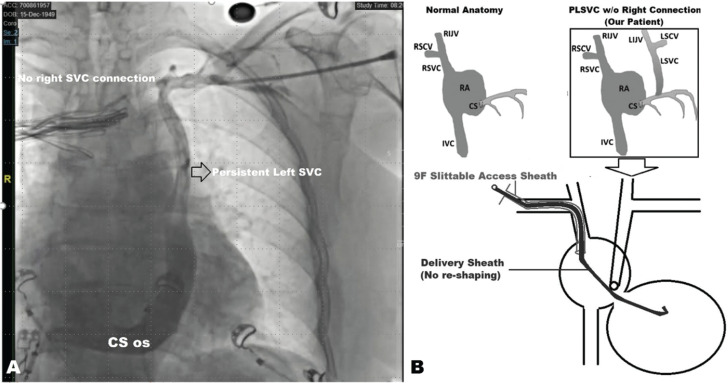
**(A)** The course of the guidewire, which was inserted through the left axillary vein puncture, was different, and the venography revealed the diagnosis of persistent left superior vena cava without a right-sided connection. **(B)** Stylet-driven ventricular lead penetrated the mid-interventricular septum with the help of a delivery sheath over a 9-Fr splittable access sheath. *Abbreviations:* CS, coronary sinus; IVC, inferior vena cava; LIJV, left internal jugular vein; LSCV, left superior cardinal vein; LSVC, left superior vena cava; os, ostium; PLSVC, persistent left superior vena cava; RA, right atrium; RIJV, right internal jugular vein; RSCV, right superior cardinal vein; RSVC, right superior vena cava; SVC, superior vena cava.

**Figure 3:**
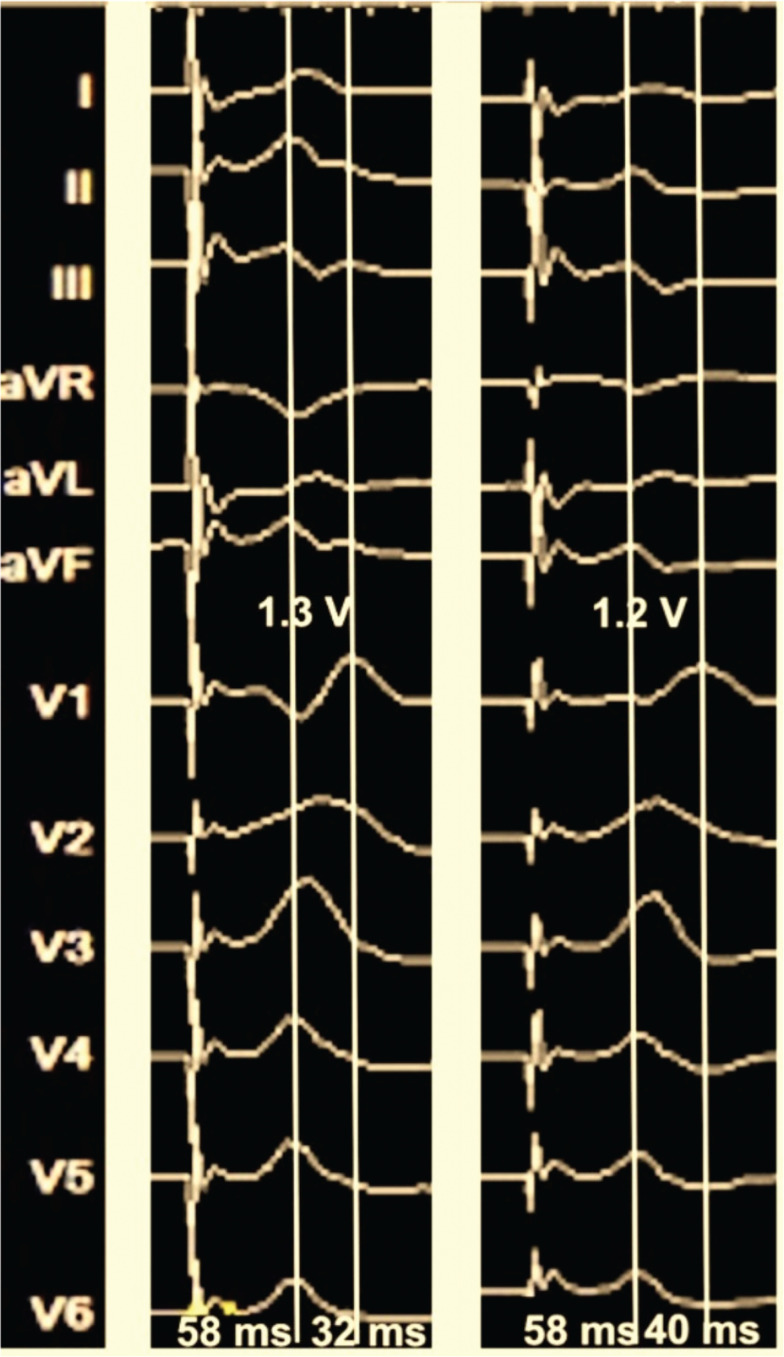
A threshold test with unipolar pacing revealed a QRS morphology transition from non-selective to selective left bundle branch pacing (no change in the V6 R-wave peak time and an increase in the V6–V1 inter-peak delay with a morphology change in the V1 terminal R-wave).

**Figure 4:**
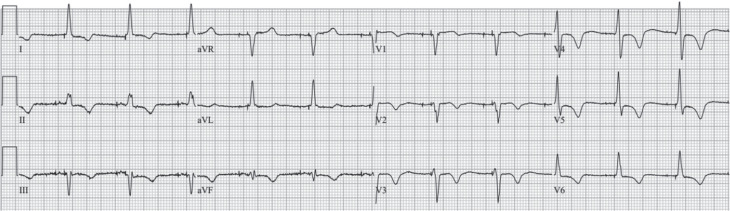
The paced QRS duration was measured as 120 ms in the post-procedural electrocardiogram.

On the third day of hospitalization, the patient was compensated after intravenous furosemide infusion. The TTE after electrical cardioversion demonstrated both septal flash and apical rocking motion besides an LVEF of 31% **(Video 1B)**. CRT via LBBAP implantation was decided during the index hospitalization.^9^ Under deep sedation and local anesthesia, a left axillary vein puncture was performed before the pacemaker pocket incision. However, the guidewire course was different, and the venography revealed the diagnosis of PLSVC without a right-sided connection **(Figure 2A**, **Video 2)**. Thus, we preferred the right-sided approach for CIED implantation. Right-sided venography revealed a normal venous anatomy. After the right axillary venous access, a DF-4 implantable cardioverter-defibrillator (ICD) lead (Biotronik, Berlin, Germany) was placed into the right ventricular apex. Then, a Solia S60 stylet-driven ventricular lead was penetrated into the mid-interventricular septum with the help of a Selectra 3D 55-39 (Biotronik) delivery sheath over a 9-French (Fr) splittable access sheath **(Figure 2B)**, and unipolar pacing parameters were compatible with non-selective left bundle branch pacing (LBBP) (a V6 R-wave peak time of 58 ms, a V6–V1 inter-peak delay of 40 ms, and a QRS transition during the threshold test) **(Figure 3, Video 3)**.^10^ Before slitting the delivery sheath, we placed the atrial lead into the right atrial appendage accordingly. The delivery sheath was slit successfully, and the sleeves fixed the electrodes. A CRT-defibrillator (CRT-D) device via LBBAP was implanted **(Video 4)**. The LBBAP lead was inserted into the LV socket, and the device was programmed to LV-only pacing mode with bipolar pacing polarity. Post-procedural echocardiography revealed that the LBBAP electrode was in the LV subendocardial region **(Video 5)**. The paced QRS duration was measured as 120 ms in the post-procedural ECG **(Figure 4)**.

**Video 2:** The guidewire’s course was different, and the venography revealed the diagnosis of PLSVC without a right-sided connection.

**Video 3:** The Solia S60-style-driven ventricular lead penetrated the mid-interventricular septum with the help of Selectra 3D 55-39 (Biotronik, Berlin, Germany) delivery sheath over a 9F splittable access sheath.

**Video 4:** A CRT-D device via LBBaP was implanted (RAO and LAO views).

**Video 5:** Postprocedural echocardiography revealed that the LBBaP electrode was in the LV subendocardial region.

We recommended AF ablation to the patient, but she declined. As a result, we administered amiodarone treatment during the follow-up. The remaining hospital stay of the patient was uneventful, and she was discharged with optimal medical therapy. The patient was asymptomatic (New York Heart Association class 1) at the first and third month outpatient visits. In the third month’s visit, echocardiography showed an LV end-diastolic diameter (LVEDD) of 51 mm, an LVEF of 46%, moderate mitral and tricuspid regurgitation, and a systolic pulmonary artery pressure of 30 mmHg.

The patient was given detailed information about how the case report could contribute to the literature. The patient provided verbal and written informed consent for the publication of this case report.

## Discussion

In our case, we performed a right-sided CRT-D implantation via LBBAP in a patient with HFrEF due to non-ischemic cardiomyopathy, whose symptoms continued despite optimal medical treatment. On the contrary, a PLSVC without a connection with a right superior vena cava (SVC) was detected incidentally during the device implantation, and a right-sided approach for CIED implantation was preferred. The presence of PLSVC usually has no clinical importance other than predicting difficulties experienced during the CIED implantation and producing alternative solutions.^11^

As the results of recent studies begin to emerge, the application of LBBAP, which provides physiological pacing as an alternative to conventional biventricular CRT, is increasing.^12,13^ As LBBAP is a newly developed method, data for handling possible difficult situations are limited. When we examined the literature, the cases mentioned were applied with a left-sided approach, and, as in our case, there was PLSVC, and few instances of LBBAP with a right-sided approach were reported. PLSVC, mainly an incidental diagnosis and a component of double SVC, is reported as the most common venous anomaly among patients undergoing CIED implantation.^14^ Akdis et al.^15^ reported the challenges and pitfalls of conventional CRT-D implantation in six patients with PLSVC. The LBBAP delivery sheath is designed for the left-sided approach, and right-sided approaches may require modification and manipulation of available tools.

A recent case report by Bulava et al.^16^ presented a left-sided approach (via the coronary sinus) for LBBAP in a patient with PLSVC without a right brachiocephalic vein connection. They penetrated the Solia S60 lead into the interventricular septum using two different sizes of delivery sheaths because of the lack of adequate contact. In another case report, McGee et al.^17^ successfully performed LBBAP via an anatomical connection between PLSVC and the right brachiocephalic vein, reportedly present in 30% of cases. However, our patient had no connection between PLSVC and the right brachiocephalic vein. We also thought that ICD and LBBAP lead implantation would be complex via the PLSVC. Thus, we planned to perform the procedure from the right side. Studies have reported the results of the right-sided approach for LBBAP, and some drawbacks of the available tools were defined. Ashur et al.^5^ reported 12 patients and Ponnusamy et al.^7^ reported 16 patients who underwent LBBAP via a right-sided approach using a C315 delivery sheath. The fluoroscopy time was longer, and the requirement for delivery sheath exchange was more frequent with the right-sided approach than with the left-sided one. A more prominent and acute angle between the right subclavian vein and the SVC is the main drawback for the right-sided approach in CIED implantation, causing kinking of the delivery sheaths, particularly for LBBAP. To prevent kinking of the delivery sheaths, a shorter splittable access sheath can preferably be chosen that is larger than the delivery sheath for LBBAP (eg, a 7-Fr splittable access sheath for the C315 delivery sheath or a 9-Fr splittable access sheath for the Selectra 3D delivery sheath).

Additionally, the appropriate acute angle around the estimated level of the right subclavian vein–SVC junction can be given to the delivery sheath (with the dilator inside the sheath) after determining the estimated distance from the right axillary vein to the septum under fluoroscopy (re-shaping method).^6^ Despite similar LBB area capture rates with the right-sided approach, the support of the delivery sheath for adequate contact with the interventricular septum was reported to be weak. Thus, specialized tools adapted for the right-sided approach are required for better results. We used a 9-Fr splittable access sheath in our patient to prevent kinking of the delivery sheath for LBBAP at the junction between the right subclavian vein and SVC. Therefore, we did not face difficulty maneuvering the delivery sheath to make contact with the interventricular septum. Furthermore, more angles or turns on the delivery sheath may cause deformation on the body of the sheath and make it hard to transmit the torque from the body to the lead helix. Thus, we did not reshape the delivery sheath. Additionally, as the distance to the target area in the septum is shorter with the right subclavian approach than with the left subclavian approach, this factor should also be considered when selecting the sheath size. Furthermore, AF predisposes to the development and decompensation of HF (chicken–egg paradox).^18^ As our patient was diagnosed with new-onset AF and the diagnosis of HFrEF had been made previously, we did not consider the AF to be the underlying etiology for HFrEF. We considered new-onset AF as the cause of the patient’s admission to the emergency department, leading to decompensated HF. However, as AF and HF would affect each other bidirectionally, we planned cardioversion during index hospitalization because of the small left atrial diameter. In a multicenter study,^19^ a simple four-parameter Antwerp score (QRS >120 ms [2 points], known etiology [2 points], paroxysmal AF [1 point], severe atrial dilation [1 point]) was validated in predicting LVEF recovery after AF ablation in patients with HF and discriminated clinical outcomes. In the Catheter Ablation Versus Medical Rate Control in Atrial Fibrillation and Systolic Dysfunction (CAMERA-MRI) trial,^20^ restoring sinus rhythm with catheter ablation resulted in significant improvements in LVEF, particularly in the absence of ventricular fibrosis on cardiac magnetic resonance imaging. The smaller LVEDD (<59 mm) and the low-voltage zones in the left atrium were also significant predictors of LVEF recovery after AF ablation.^21^ Our patient’s Antwerp score was 2 points (QRS duration >120 ms), and the LVEDD was small. Thus, the probability of LVEF recovery was high based on the previous data.^19,21,22^ As in our patient, the combination of sinus rhythm and true LBBB increases the success rate of LBBAP.^23^ Besides CRT-D implantation, all those positive outcomes of restoring sinus rhythm in our patient might help improve LVEF.

The optimal timing for CRT implantation in HFrEF remains unclear. In a previous study, Leyva et al.^9^ showed that delays from a first HF hospitalization (HFH) to CRT implantation were associated with progressively worse long-term clinical outcomes. The best outcomes after CRT were observed in patients with no previous HFHs and in those undergoing implantation during their first HFH. After conversion to sinus rhythm, our patient had prolonged first-degree atrioventricular block (a P–R interval of 240 ms) and LBBB (a QRS duration of 160 ms). Therefore, we preferred to implant a CRT during the index HFH. However, there is an endless debate in implanting a CRT-pacemaker (CRT-P) versus a CRT-D in such patients, particularly for non-ischemic HF.^24^ Based on the previous data, there is still no solid evidence of greater survival benefit by CRT-D compared with CRT-P in patients with a clinical indication of CRT.^25^ Nonetheless, it is very clear from the CRT Survey II that doctors across the European Society of Cardiology (ESC) perceive CRT-Ds as preferable.^26^ We also chose the CRT-D for our patient after discussing the choices with her because of lower comorbidities, despite her non-ischemic etiology, atrial fibrillation, older age, and female sex.

In conclusion, LBBAP has emerged as an essential alternative to conventional CRT-D implantation in clinical practice. The clinical applicability of LBBAP is increasing, with well-documented outcomes. As the number of LBBAP increases, the operators will face challenges, such as PLSVC. Right-sided LBBAP can be successfully performed with existing equipment. However, further studies and specified tools are needed to increase the success rates of the procedures with lower procedural and fluoroscopy durations.
